# Retinoic acid receptor regulation of decision-making for cell differentiation

**DOI:** 10.3389/fcell.2023.1182204

**Published:** 2023-04-04

**Authors:** Geoffrey Brown

**Affiliations:** School of Biomedical Sciences, Institute of Clinical Sciences, College of Medical and Dental Sciences, University of Birmingham, Birmingham, United Kingdom

**Keywords:** retinoic acid receptors, vitamin A, stem cells, differentiation, haematopoiesis

## Abstract

All-*trans* retinoic acid (ATRA) activation of retinoic acid receptors (RARs) is crucial to an organism’s proper development as established by findings for mouse foetuses from dams fed a vitamin A-deficient diet. ATRA influences decision-making by embryonic stem (ES) cells for differentiation including lineage fate. From studies of knockout mice, RARα and RARγ regulate haematopoiesis whereby active RARα modulates the frequency of decision-making for myeloid differentiation, but is not essential for myelopoiesis, and active RARγ supports stem cell self-renewal and maintenance. From studies of zebrafish embryo development, active RARγ plays a negative role in stem cell decision-making for differentiation whereby, in the absence of exogenous ATRA, selective agonism of RARγ disrupted stem cell decision-making for differentiation patterning for development. From transactivation studies, 0.24 nM ATRA transactivated RARγ and 19.3 nM (80-fold more) was needed to transactivate RARα. Therefore, the dose of ATRA that cells are exposed to *in vivo*, from gradients created by cells that synthesize and metabolize, is important to RARγ *versus* RARα and RARγ activation and balancing of the involvements in modulating stem cell maintenance *versus* decision-making for differentiation. RARγ activation favours stemness whereas concomitant or temporal activation of RARγ and RARα favours differentiation. Crosstalk with signalling events that are provoked by membrane receptors is also important.

## Introduction

Vitamin A is required for the early development of many organs, including the eye, forelimbs, heart, hindbrain, posterior body axis, somites, and spinal cord (Clagett-Dame and Knutson, 2011; Berenger and Duester, 2022). ATRA, the most active metabolite of vitamin A, controls gene expression *via* the transcriptional activation of the three RAR types RARα, RARβ, and RARγ. They, as a heterodimer with retinoid X receptor, bind to target gene retinoic acid response elements (RAREs) to drive transcription when ATRA binds to RARs. When ATRA is absent, DNA-bound RAR/RXR heterodimers are associated with corepressors which with recruited histone deacetylases maintain a condensed chromatin and repress gene expression (Chambon, 1996).

A key question was does the transcription action of ATRA depends entirely on RARs? To answer, investigators developed RARα, RARβ, and RARγ triple knockout murine embryonic stem (ES) cell lines and examined genome-wide transcription following treatment with ATRA for 24 and 48 h (Laursen and Gudus, 2018). ATRA did not affect the genome-wide transcriptional profile of the triple knockout ES cells. By contrast to wild type cells, proliferation was not affected by ATRA, the induction of differentiation markers (Cyp26a1, Hoxa1, Cdx1, Stra8, CoupTF1 and Meis1) was abrogated, and the repression of stem cell markers (Nanog, Oct4, Zfp42, Sox2, Klf4, and Sall4) was perturbed. *Cyp26a1* and *Hoxa1* are RARγ target genes (Kashyap et al., 2011; Kashyap et al., 2013) and ATRA failed to induce their expression within RARγ knockout and RARβ and RARγ double knockout cells (Laursen and Gudus, 2018). The transcriptional effects of ATRA and the capacity to decrease pluripotency markers, induce differentiation, and drive growth arrest are dependent on RARs. Even so, the biological functions of vitamin A and ATRA include non-genomic effects (Tanoury et al., 2013).

This review examines the roles of RARs in stem cell decision-making for cell differentiation from information gained from knockout mice and *in vitro* studies of ES cells and haematopoietic stem cells (HSCs). Regarding HSCs, various findings have challenged a longstanding model for how HSCs ‘choose’ to differentiate towards a mature cell type. Though new principles are still a matter of debate they are highly pertinent to consideration of how RARs influence HSC decision-making. In the conventional model of haematopoiesis, the progeny of HSCs undergoes a series of stepwise commitment decisions that eventually restrict intermediate progenitors to a single cell lineage. But HSCs can affiliate directly to a cell lineage as they are a heterogeneous population of cells as evidenced by the existence of megakaryocyte, erythroid, and macrophage lineage biased/affiliated HSCs [reviewed in Brown, 2020]. HSCs “choose” to develop along a pathway from a continuum of all options (Ceredig et al., 2009). Their differentiation is a continuous process that lacks a definite point of commitment (Velten et al., 2017) because trajectories are broad and flexible and HSCs and progenitors that have adopted a lineage fate can still veer towards an alternative fate (Nestorowa et al., 2016). Cytokines play a key role in orchestrating lineage affiliation because erythropoietin and macrophage colony stimulating factor instruct erythroid and myeloid fate within HSCs, respectively (Grover et al., 2014; Mossadegh-Keller et al., 2013). Granulocyte colony-stimulating factor and macrophage colony-stimulating factor instruct granulocyte and macrophage fate within bipotent progenitors, respectively (Rieger et al., 2009; Metcalf and Burgess, 1982). Crosstalk between ATRA- and membrane receptor-provoked events has been known for some time from the negative cross-modulation between RARs and the activator protein 1 (AP-1), which regulates gene expression in response to cytokines (Nicholson et al., 1990). AP-1 has been implicated in the regulation of the erythropoietin-driven survival/proliferation of erythroid cells (Jacobs-Helber et al., 1998).

## Findings from studies of the development of embryonic cells

Findings for RAR null mutant mice confirmed the importance of vitamin A to embryonic development and addressed whether RARs are essential transducers of ATRA signalling *in vivo*. The defects seen for double null mutant mice recapitulated the congenital malformations seen in foetuses from dams fed a vitamin A-deficient diet, and these null mutant mice displayed additional abnormalities (Lohnes et al., 1994). Deletion of the whole RARα led to death of >90% of the homozygotes before the age of 2 months, but the mice failed to display any of the vitamin A deficiency-associated lesions other than testis degeneration. Mice null for the predominant RARα1 isoform appeared to be normal (Lufkin et al., 1993). Mice lacking all forms of RARβ developed normally (Luo et al., 1995), and mice lacking the most abundant RARβ2 isoform appeared to be normal (Mendelsohn et al., 1994). Mice null for all isoforms of RARγ exhibited deficient growth, early lethality, squamous metaplasia of the seminal vesicles and prostate, and male sterility, and RARγ2 null mice appeared to be normal (Lohnes et al., 1993). There is a degree of functional redundancy among the RARs. In keeping, RARγ null-cells failed to differentiate *in vitro* in response to ATRA and express several ATRA-induced genes and re-expression of RARγ or overexpression of RARα restored differentiation potential and target gene activation. RARβ restored target gene activation but poorly restored differentiation potential. ATRA-activation of *Cdx1*, *Gap43*, *Stra4*, and *Stra6* was specifically impaired within RARγ-null cells pointing to a distinct subset of target genes for RARγ and for other RARs (Taneja et al., 1995).

Embryonic stem (ES) cells are competent regarding the production of all the cell types and ATRA has been used to obtain different cell types from mouse ES cells grown as monolayers or as hanging drops to form embryoid bodies. Cells resembling male germ cells spontaneously arise from embryoid bodies and treatment with 2 μM ATRA, with or without testosterone, significantly increased the expression of male germ cell lineage-associated genes. (Silva et al., 2009). The findings for the generation of neuronal cells vary according to the cells and conditions used. ATRA activates transcription of the *Hoxa1* gene in ES cells and monolayer cultures of Hoxa1^−/−^ ES cells treated with ATRA expressed genes that are associated with embryonic brain development at a lower level than wild type cells. The reintroduction of exogenous Hoxa1 was needed for 5 μM ATRA-induced ES cell neuronal differentiation (Martinez-Ceballos and Gudas, 2008). Other investigators reported that 1 μM ATRA enhanced the efficiency of differentiation of ES cells into neural precursor cells (Li et al., 2019) and that this conversion required both fibroblast growth factor and the elimination of signals for other fates (Ying et al., 2003). Neural precursor cells have been efficiently derived from ES cells without the addition of ATRA by using a three-dimensional culture system followed by two-dimensional derivation. The investigators used fibroblast growth factor and the B27 medium supplement containing retinyl acetate, which is a natural form of vitamin A (the acetate ester of retinol) and a precursor in ATRA metabolism (see below regarding the importance) (Yoon et al., 2021). A 100 nM concentration of ATRA has been used to induce the expression of mesodermal marker genes within mouse ES cells (Oeda et al., 2013).

The use of a pharmacological amount of ATRA (1—5 μM) to differentiate ES cells is a concern. The affinities of ATRA for RARα, RARβ, and RARγ are 9 nM, 3 nM, and 10 nM, respectively (Idres et al., 2002), and the physiological concentration of ATRA in tissues is ∼1—10 nM (Czuba et al., 2020). ES cells are usually cultured in medium plus either foetal calf serum or the B27 supplement (Amit and Itskovitz-Elder, 2002; Sadhananthan and Touson, 2005) and they differentiate into cells that arise from the three germ cells layers. Medium with 10% foetal calf serum contains 50 nM all-*trans*-retinol and the level of ATRA in the serum of humans and other mammalians is ∼ 4–14 nM (Baltes et al., 2004). As mentioned above, the B27 supplement contains retinyl acetate and ES cells cultured in the B27 supplemented medium synthesized ATRA and their differentiation towards neural precursor cells (expressing *Sox1*) was reliant on ATRA produced endogenously (Engberg et al., 2010). ATRA was not measurable and to demonstrate the need for neuronal differentiation the investigators either removed retinyl acetate from the B27 supplemented medium, inhibited the enzymes that catalyse the synthesis of ATRA, or used the pan-RAR antagonist AGN193109 to block the activity of RARs and neural differentiation was prevented. For retinyl acetate deprived cells, neuronal differentiation was restored by the addition of 1 nM ATRA. When ATRA signalling was inhibited, there was change of fate from neuronal to mesoderm and Nodal signalling had repressed neuronal development in a Wnt-dependent manner. The investigators concluded that a neuronal to mesoderm fate switch depends on active Nodal-, Wnt-, and FGF signalling. It is well-documented that signalling *via* Nodal, a transforming growth factor β-related factor, and Wnt glycoproteins stimulation of complex intracellular signalling cascades play roles during organogenesis and ES cell differentiation (Schier and Shen, 2000; Takenaga et al., 2007; Koyima and Habas, 2008; Sokol, 2011).

The roles of individual RARs within mouse ES cells have been examined by homologous recombination disruption of the *Rara* and *Rarg* genes (Tanoury et al., 2014). ES cells were cultured as cellular aggregates and treated with 2 μM ATRA to generate neuronal cells. ES cells lacking RARα became neural progenitors, giving rise to neurons, cells lacking RARγ failed to do so, and RARγ2 rescued lines gave rise to neurons pointing to a role for RARγ2 in ES cell neuronal development. From comparison of ATRA-induced gene expression by wild type and the RARγ2 restored cells and RT-qPCR experiments, the investigators showed that RARγ2 regulates a small number of genes, exemplified by *meis homeobox 2* *(Meis2)*, *left right determination factor 1* (*Lefty1*), *hepatocyte nuclear factor 1 homeobox B* (*Hnf1b*), *arginase 1*, and the homeobox (Hox) genes *Hoxa3*, and *Hoxa5*. RARs are phosphorylated in response to ATRA and the importance of phosphorylation of RARγ2 was investigated by expressing forms of RARγ2 that had been modulated in phospho-acceptor sites. For ATRA-mediated neuronal differentiation, gene expression that was controlled by phosphorylation of RARγ2 included that of *Meis2*, *Lefty1*, *Hnf1b*, and *gastrulation brain homeobox 2*. The genes targeted by phosphorylated RARγ2 depicted atypical DR7 retinoic acid response elements in addition to canonical DR2 and DR5 elements and the phosphorylated form of RARγ2 was recruited by DR7 and DR5 elements in response to ATRA. RARα1 was phosphorylated *in vitro* and *in vivo* by protein kinase A and phosphorylation at the site is involved in dibutyryl cAMP modulation of the differentiation of F9 embryonal carcinoma cells (Rochette-Egly et al., 1995).

Lessons from studies of ES cells are as follows. RARγ2 plays a role in ATRA-induced ES cell neuronal differentiation by either regulating the expression of specific genes or closing “unwanted” options regarding a proposed need. A physiological level (nM) of ATRA influences decision-making for differentiation including lineage fate. Endogenously produced ATRA plays a key role because vitamin A signalling is, in essence, driven by the intracellular ATRA concentration. In general, evaluation of the effect of treating cells with ATRA is confounded by all-*trans*-retinol or retinyl acetate in medium (Czuba et al., 2020) because of the need to take into consideration cryptic ATRA signalling from endogenous synthesis. Moreover, when epidermal keratinocytes were cultured in medium supplemented with 5% foetal calf serum, which contained 25 nM all-*trans* retinol and ATRA was undetectable, the level of all-*trans* retinol led to an intracellular level of ATRA of 25—50 nM (Randolph and Simon, 1997). This is well within the range of ATRA for activation of RARs. Growth factor-provoked signalling events are important to ES cell differentiation and RAR phosphorylation allows the relay of information from cell-surface receptor-provoked kinase cascades. Additionally, ATRA provoked non-transcription effects within differentiating mouse embryonic stem cells include the rapid and transient activation of kinase cascades [reviewed in Rochette-Egly, 2015].

## Findings for RARα and haematopoietic cell differentiation

The RARα gene is expressed in almost all adult tissues, and expression of the major isoform RARa1 is also ubiquitous (Leroy et al., 1991). During haematopoiesis, RARα is expressed by HSCs and their differentiating offspring. Mouse lineage-negative, c-kit–positive, Sca-1–positive (LKS+) cells, that contain HSCs, and lineage-negative, c-kit–positive, Sca-1– negative (LKS−) cells, that lack HSCs, expressed RARα (Purton et al., 2006). RARα, particularly RARα2, expression increased dramatically during myeloid differentiation as seen for the induced differentiation of FDCP mixA4 mouse progenitor cells (Zhu et al., 2001). In addition to controlling homeostasis, RARs control the functional activity of some of the mature blood cells regarding the production of inflammatory cytokines [reviewed in Duong and Rochette-Egly, 2011]

A role for RARα in myeloid differentiation is well established (Collins, 2002). ATRA promotes the differentiation of promyeloid cell lines and normal myeloid progenitors and findings for the human promyeloid cell line HL-60 established a role for RARα in neutrophil differentiation. HL-60 cell differentiate towards neutrophils in response to treatment with ATRA (Breitman et al., 1980) and macrophages when treated with 1α,25-dihydroxyvitamin D3 (1,25D) to activate the vitamin D receptor (VDR) (Mangelsdorf et al., 1984). HL-60 cells undergo a low rate of spontaneous neutrophil differentiation because around 3%–10% of cells are more mature myelocytes, metamyelocytes, and banded and segmented neutrophils. A 1 μM concentration of ATRA promoted neutrophil differentiation with 90% of the cells terminally maturing, and 100 nM was effective. The identification of a PML-RARα fusion transcript in the cells from patients with acute promyelocytic leukaemia (de The et al., 1990) focused attention on RARα playing a key role during HL-60 neutrophil differentiation. Retroviral vector-mediated transduction of a single copy of RARα into an ATRA-resistant HL-60 subclone restored ATRA sensitivity for differentiation (Collins et al., 1990). Similarly, RARα agonism, by using AGN195183, was sufficient to promote HL-60 cell differentiation towards neutrophils (Brown et al., 2017). As mentioned above, G-CSF directs normal granulocyte/macrophage progenitors towards neutrophils (Rieger et al., 2009) and co-operates with ATRA to promote HL-60 differentiation towards neutrophils. Treatment of HL-60 cells with 10 nM ATRA led to a low level of neutrophil differentiation, differentiation was rapid and effective when 10 nM ATRA was combined with 30 ng/ml G-SCF, and G-CSF alone had no effect. A low dose of ATRA had rendered HL-60 cells responsive to the action of G-CSF (Sakashita et al., 1991; Bunce et al., 1994).

The influence of ATRA on neutrophil differentiation is particularly well-documented. ATRA and RARα also promote monocyte differentiation as shown from studies of the promyelocytic cell line NB4 and myeloblast blast-like cell line KG-1 (Brown et al., 2017). These cells express RARα and do not express RARγ. For NB4cells, 100 nM of the RARα agonist promoted neutrophil differentiation (40% CD11b+/CD14-ve cells) and treatment with 10 nM 1,25D led to a low level of monocyte differentiation (CD11b+/CD14+ve and ∼6%). The combined use of 100 nM of the RARα agonist and 10 nM 1,25D increased the level of monocyte differentiation (to ∼50%). KG-1 cells differentiated towards neutrophils to a very small extent (∼5%) in response to 100 nM of the RARα agonist and 10 nM 1,25D did not have a significant effect. Like NB4 cells, there was a significant level of monocyte differentiation (18%) when KG-1 cells were treated with the RARα agonist and 1,25D. RARs interact with several other nuclear receptors (Chambon, 1996) and the rationale to the interplay between the actions of the RARα agonist and 1,25D is that ATRA activation of RARα within NB4 and KG-1 cells upregulated the expression of a transcriptional variant of VDR that originates in exon 1a. KG-1 cells expressed RARα protein at a high level which in the absence of ATRA had repressed transcription of the VDR gene. The receptor interplay is complex and linked to cell status because ATRA downregulated VDR mRNA for HL-60 cells differentiating towards neutrophils in response to ATRA (Marchwicka et al., 2016).

For normal human bone marrow myeloid progenitor cells, ATRA supported their differentiation towards neutrophils but not towards erythrocytes (Gratas et al., 1993). Findings from *in vitro* cultures of cells from null mutant mice suggested roles for RARα1 and RARγ in neutrophil maturation (Labrecque et al., 1998). This was normal within myeloid colonies that were grown in methylcellulose from the bone marrow cells harvested from RARα1 and RARγ knockout mice. Neutrophil differentiation within myeloid colonies from cells harvested from the RARα1 and RARγ double knockout mouse was blocked at the myelocyte stage. The differentiation of cells within erythroid and macrophage colonies was not affected. The distribution of neutrophils, macrophage, and erythroid colonies was the same for cells from the wild type mice and the RARα1, RARγ, and compound null mutants, suggesting that lineage choice was not affected by a lack of RARα1 and/or RARγ. By contrast, various studies have reported that ATRA-mediated enhancement of myeloid-colony growth associates with a reduced production of colonies containing cells of other lineages, suggesting an influence of ATRA on multipotent cells [reviewed in Collins, 2002].

The influence of ATRA on primitive mouse haematopoietic cells was different to that seen for myeloid progenitor cells. Like ES cells, mouse LSK + cells, which are enriched for HSCs, differentiate spontaneously in liquid suspension culture and treatment with 1 μM ATRA delayed their differentiation. LSK + cells were allowed to differentiate for 7 days and then treated with 1 μM ATRA. The effect of ATRA on committed progenitor cells arising from the cultured LSK + cells was as seen for normal human bone marrow myeloid progenitor cells and HL-60 cells. There was a markedly decreased level of colony-forming cells and enhanced neutrophil differentiation which was attributed to enhanced maturation of committed granulocyte/monocyte progenitors (Purton et al., 1999).

Dormant mouse HSCs metabolize all-*trans* retinol to ATRA in a cell-autonomous manner (Cabezas-Wallscheid et al., 2017) and the aldehyde dehydrogenases (ALDHs), a family of oxidoreductases, convert retinaldehyde into ATRA. The importance of endogenous retinoid metabolism to cultured mouse HSCs (CD34^−^ LSK+) was investigated by using diethylaminobenzaldehyde to inhibit ALDH activity. This impeded HSC differentiation leading to a ninefold expansion of HSCs, as measured by cells that were able to reconstitute lethally irradiated mice (Muramoto et al., 2010). Targeted siRNA of ALDH1a1 in HSCs revealed that this ALDH was the target of diethylaminobenzaldehyde inhibition. Similarly, inhibition of ALDH activity led to expansion of human HSCs that were able to repopulate NOD/SCID mice (Chute et al., 2006). From these studies, ALDH regulates HSC differentiation whereby the conversion of retinaldehyde into ATRA promotes HSC differentiation. Cyp26b1 is generally viewed as an enzyme that limits the effects of ATRA on cells by metabolising ATRA to 4-oxo-retinoic acid. A recent omics analysis revealed that the maintenance of mouse HSCs was reliant on the production of 4-oxo-retinoic acid and transmission of 4-oxo-retinoic acid-mediated signalling *via* RARβ (Schonberger et al., 2022). 4-oxo-retinoic acid activates the three RARs and the level required for activation of RARβ is lower than that for RARα and RARγ (EC_50_ values of 33 nM, 8 nM, and 89 nM, respectively) (Idres et al., 2002).

Purified human lineage-, CD133+, CD34^+^ cells are enriched for HSCs and the role of RARα was investigated by treating these cells with antagonists. Cell production peaked at day 20 for control cultures and viable cells then declined rapidly. The pan-RAR antagonist AGN194310 treated cultures were maintained for up to 55 days, with 4-fold more cells by day 40. Both cultures produced mostly neutrophils and monocytes, in equal ratios, with the antagonist treated cultures generating more of the two mature cell types (as to the increased cumulative cell number) (Brown et al., 2017). AGN194310-provoked increased myeloid cell production by HSCs is in keeping with neutrophil numbers were strikingly increased in mice treated with AGN194310 (Walkley et al., 2002) and that vitamin A deficiency in mice caused a systemic expansion of myeloid cells (Kuwata et al., 2000). CD11b+ differentiated myeloid cells appeared and immature myeloid cells declined at the same rates in both control and AGN194310 treated cultures. Switching-off RARs had not slowed down myeloid cell differentiation and instead there was enhanced expansion of lineage-, CD133+, CD34^+^ cells and colony-forming progenitors within the AGN194310 treated cultures. Antagonism of RARα, by AGN195183, was sufficient for the enhanced expansion of lineage-, CD133+, CD34^+^ cells and antagonising RARγ did not lead to this enhancement. At first sight it seems paradoxical that antagonism of all RARs had delayed human HSCs differentiation and that agonism of all RARs (with ATRA) had delayed mouse LSK + cell differentiation (see above). The two cell populations are different regarding their heterogeneity and the culture conditions used were different. Otherwise, the findings point to complex actions for RARα and RARγ. A lack of active RARα (RARα antagonism) had delayed the differentiation of human lineage-, CD133+, CD34^+^ cells whereas the presence of activated RARγ (ATRA agonism) may have interfered with the differentiation of the mouse LSK + cells (see also later).

From studies of haematopoiesis, active RARα plays a role to enhance neutrophil differentiation of mouse progenitor cells and human HSCs and promyeloid cell lines. The role of RARα is to modulate/regulate rather than being essential for myelopoiesis (Kastner and Chan, 2001). For example, the terminal differentiation of cultures of human HSCs towards neutrophils and macrophages was unaffected when RARα was antagonised. For these cells, antagonism of RARα did not alter the relative proportions of neutrophils *versus* macrophages generated nor appeared to accelerate the terminal maturation of immature myeloid cells. Instead, active RARα positively modulated the frequency of decision-making for differentiation to favour differentiation. In other words, unliganded interferes with gene expression for differentiation which is promoted when ATRA or a specific RARα agonist is bound (Kastner and Chan, 2001; Collins, 2002). G-CSF is a key regulator of granulopoiesis and, like RARα, was dispensable. The combined action of these agents was investigated by conditional deletion of RARα on a G-CSF receptor-null background and treating G-CSF receptor null mice with the pan-RAR antagonist AGN194310 (referred to as NRX194310); granulopoiesis persisted in these mice (Chee et al., 2013). To differentiate or not is a multifactorial decision and the following section examines the extent to which RARγ plays a role in decision-making for cell differentiation.

## Findings for RARγ and cell differentiation

Unlike RARα, the distribution of RARγ is very restricted and specific spatial and temporal distributions of RARγ during mouse embryogenesis led to the proposal that RARγ plays a role in early morphogenic events (Ruberte et al., 1990). During haematopoiesis, RARγ is selectively expressed by hematopoietic stem cells and primitive progenitors (Purton et al., 2006). In keeping with a restricted expression of RARγ within primitive cells, the binding sites for RAR/RXR dimers within undifferentiated F9 embryonal carcinoma cells coincided with loci that are targeted by transcription factors that are important to pluripotency (SOX2, NANOG, and POU5f1) (Chatagnon et al., 2015).

A special consideration to the role of RARγ *versus* that of RARα is the level of ATRA that cells are exposed to influences whether RARγ or RARγ together with RARα are transactivated within cells. The concentration of ATRA that is needed to activate RARγ is substantially lower than that required for activation RARα. A 0.24 nM level of ATRA transactivated RARγ whereas 19.3 nM (80-fold more) was needed to transactivate RARα (Brown et al., 2017). By contrast, the best ATRA induction of transcription was obtained for RARα which was 10-fold higher than that for RARγ, as seen from the fold-induction of luciferase activity from a RARE-tk-Luc reporter plasmid in the presence of each RAR (Idres et al., 2002). The increased fold induction by RARα suggests a greater efficiency to inducing transcription as governed by the interaction of the ligand-activated receptor complex with response elements.

The nature of the genes that are regulated by RARγ is germane to consideration of a role for RARγ. Comparison of ATRA-induced events within the wild type and RARγ null ES cells showed that RARγ is essential for ATRA-induced epigenetic marks at gene promoters, chromatin remodelling, and transcriptional activation. ATRA activation of RARγ greatly increased the transcript levels of genes that encode regulators of ATRA metabolism within cells. They were the genes encoding stimulated by retinoic acid 6 (Stra6), lecithin:retinol acyltransferase (LRAT), cellular retinoic acid binding protein 2 (CRABP2), and cytochrome p450 26A1 (CYP26A1) (Kashyap et al., 2013). Retinol-binding protein 4 (RBP4) transports all-*trans* retinol (vitamin A) in the blood for transfer into cells which is mediated by binding to Stra6. LRAT converts all-trans retinol into retinyl esters for storage, CRABP2 delivers ATRA to the nucleus and RARs, and CYP26A1 catabolises ATRA to polar metabolites for elimination. RARγ expression by stem cells might autoregulate a low ATRA content by virtue of ATRA activation leading to the diversion of “excess” all-*trans* retinol into retinyl esters for storage and elevated CYP26A1 expression increasing ATRA breakdown. These controls on ATRA-driven RAR events may be an important “housekeeping” function to stem cell stemness. From the studies of ATRA-regulated genes in the early zebrafish embryo, CYP26A1 was identified as one of the most robust genes regarding ATRA regulation, even when ATRA availability is drastically reduced (Samarut et al., 2014), and, as above, RARγ is transactivated by sub nM ATRA. A proposal from the zebrafish studies was also that RARγ subtypes appeared to play roles in the basal regulation of ATRA-responsive gene expression with RARα subtypes playing roles in the transcriptional response of cells to ATRA. (Samarut et al., 2014).

The above considerations point to active RARγ promoting stem cell maintenance and stemness. Indeed, RARγ plays a critical role in balancing HSC self-renewal/maintenance *versus* differentiation (Purton et al., 2006). The bone marrow of RARγ knockout mice had markedly reduced numbers of HSCs, measured as transplantable repopulating cells per femur, and the numbers of mature myeloid progenitors were increased. *Ex vivo* activation of RARγ, by ATRA, promoted the self-renewal of HSCs because loss of RARγ abrogated the capacity of ATRA to enhance the maintenance of HSCs in culture. Regarding stem cell stemness, a much more undifferentiated phenotype was seen for primitive haematopoietic precursors that were retroviral-mediated transduced to overexpress RARγ whereas primitive precursors that overexpressed RARα differentiated predominantly to granulocytes.

As considered above, RARγ2 has a role in specifying ES cell neuronal differentiation which might relate to either a positive influence or the proposed need for the elimination of other fates (Ying et al., 2003). Studies of zebrafish embryos revealed a negative role for active RARγ in cell differentiation. RARγ transcripts are restricted to primitive cells at the later stages of the zebrafish embryo. At 24 h post fertilisation they were restricted to mesodermal and neural crest stem and progenitor cells, in the head area, in the lateral plate mesoderm, and in the pre-somitic mesoderm of the tail bud. Transcripts were still visible in the tail bud at 48 h post fertilisation (Hale et al., 2006). Zebrafish embryos were treated at 4 h post fertilisation (hpf) with 10 nM of the RARγ-selective agonist AGN205327. This dose of the agonist is close its binding affinity for RARγ (an ED_50_ of 32 nM). Treatment led to the development of viable fish that were substantially abnormal. There were substantial changes to head morphology, associated with loss of cranial bones and tissue, a reduced antero-posterior axis length, due to somite loss, and heart abnormalities that led to oedema. Regarding the loss of cranial bones and anterior line ganglia, the prevalence of Sox-9 neural crest cells was not affected other than a slight decrease in the head region. The agonist prevented caudal and pectoral fin formation and Tbx5a progenitors that form the pectoral fin were present in the bud region. The lack of pectoral outgrowth, provoked by the RARγ agonist at 4 hpf, was reversed by the addition, at 23 h post fertilization, of a RARγ antagonist, to reverse the action of the RARγ agonist, or wash out of the RARγ agonist at 23 h. The experiments were performed in E3 medium (for zebrafish embryos) which is a balanced salt solution. Therefore, and in the absence of exogenous ATRA, agonising RARγ had disrupted stem cell decision-making for differentiation for the patterning of zebrafish development. The option for fin development had been sustained within bud stem cells as to the reversibility of the action of the RARγ agonist (Wai et al., 2015). Regarding reversibility, it is noteworthy, as mentioned above, that RARγ regulates ATRA-induced chromatin epigenetic marks for gene expression within ES cells (Kashyap et al., 2013).

## Controls on stem cell decision-making

**FIGURE 1:**
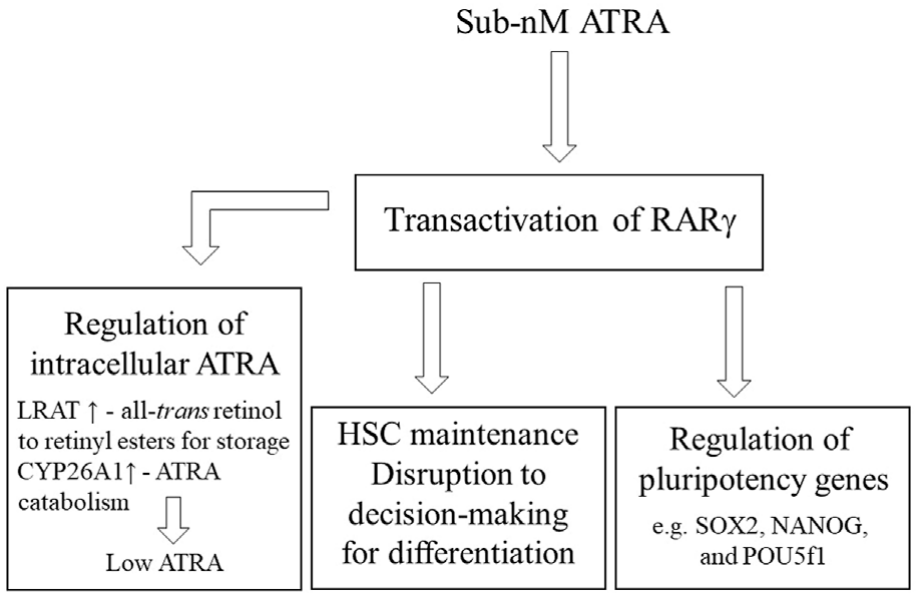
Controls on stem cell stemness Active RARγ is required for the maintenance of HSCs, for zebrafish embryos activation disrupted stem cell decision-making for differentiation for the patterning of development, and RARγ plays a role in regulating pluripotency genes. Genes that regulate the intracellular level of ATRA are expressed upon RARγ transactivation whereby lecithin:retinol acyltransferase (LRAT) converts all-*trans* retinol to retinyl esters for storage and cytochrome p450 26A1 (CYP26 A1) catabolizes ATRA. A sub nM intracellular level is sufficient to transactivate RARγ.

The maintenance of stem cells is crucial to an organism to meet the need to replace any damaged and worn-out cells throughout life. The controls that ensure a pool of stem cells are likely to be multiple, and, therefore, rigorous. The presence of active RARγ regulates the maintenance of stem cell stemness because pluripotency genes are direct targets of RARγ. A sub-nM concentration of ATRA is sufficient for transactivation of RARγ. A higher level of ATRA is needed to transactivate RARα, to favour decision-making for differentiation. Stem cells may protect themselves from differentiation that is favoured by RARα activation, by a higher level of ATRA, by means of RARγ-mediated upregulation of the expression of LRAT, for the storage of all-*trans* retinol, and CYP26, for the degradation of ATRA (Figure 1). Regarding a low intracellular level of ATRA within stem cells, it has been proposed that ES cells do not have all the enzymes that are needed to metabolise all-*trans* retinol into ATRA (Chen and Khillan, 2010; Khillan, 2014). Instead, all-*trans* retinol has been proposed to play a role in promoting the self-renewal of ES cells by the direct activation of the phosphoinositide 3 kinase/Akt signalling pathway *via* insulin-like growth factor-1.

ATRA was used at a dose that activates RARα and RARγ for *in vitro* differentiation of the cells considered above. Studies have examined the effect of treating P19 and F9 embryonal carcinoma cells with a combination of the synthetic RARα- and RARγ-selective agonists Am80 and CD666, respectively (Roy et al., 1995). As seen for ATRA activation of RARα and RARγ, treatment of P19 cells with non-selective concentrations of AM80 and CD666, for activation of both RARα and RARγ, induced expression of the *Stra1*, *Stra2*, *CRABPII*, *RARβ* and *Hoxa-1* genes. *Stra1*, *Stra2*, and *CRABPII* were not induced when Am80 and CD666 were used separately at a receptor-selective concentration, and the induction of *RARβ* and *Hoxa-1* was substantially reduced. The combined use of receptor-selective concentrations of AM80 and CD666 led to inductions. Similarly, P19 cells differentiated when treated with Am80 and CD666 when each agonist was used at a non-selective concentration, they failed to do so when each agonist was used at a receptor-selective concentration, and the combined use of receptor-selective concentrations of Am80 and CD666 led to differentiation The findings for F9 cells were similar other than the combinations of compounds appeared to be less efficient. The differentiation provoked by the combined use of agonists at receptor-selective concentrations was viewed as an additive/synergistic action and support to functional redundancy regarding RARα and RARγ. Alternatively, from these studies and the potent differentiating effects of ATRA the concomitant or temporal activation of both RARα and RARγ favours decision-making for differentiation in a more complex manner. Regarding complexity, RAR/RXR binding elements can distinguish pluripotency-from differentiation-associated genes which appears to be mediated by different sets of regulatory regions, with DR0-containing regions favoured in undifferentiated and DR5-enriched in differentiated cells (Chatagnon et al., 2015).

**FIGURE 2:**
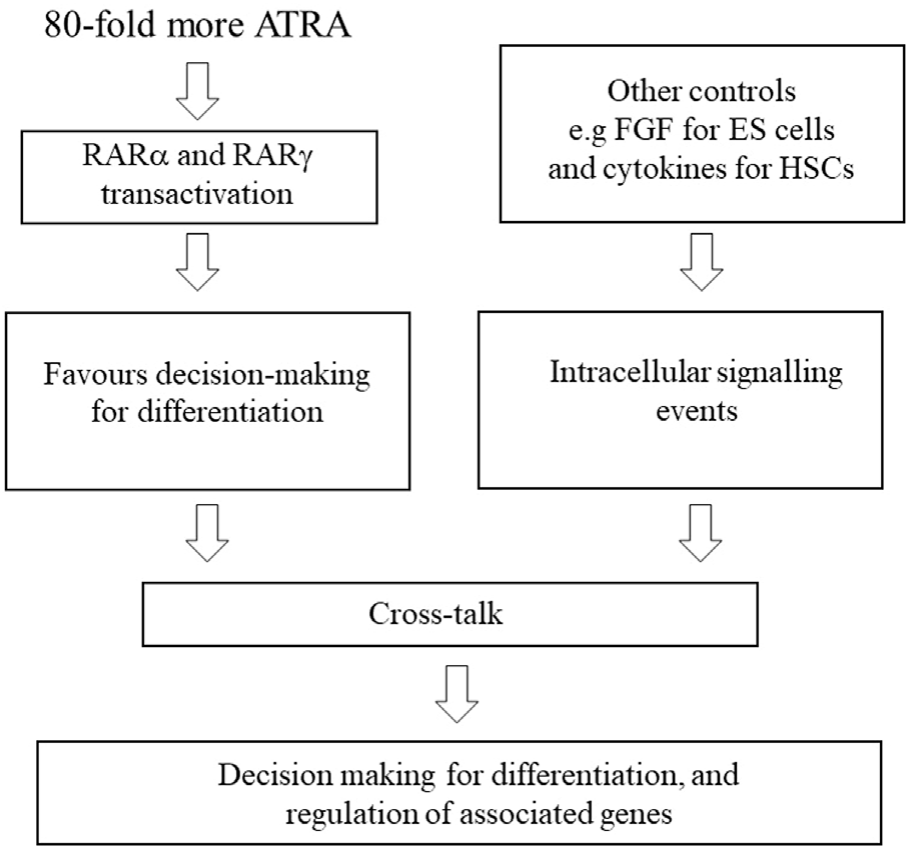
Controls on decision making for differentiation. For cell differentiation, ATRA is used at a dose that activates RARα and RARγ. As for ATRA, concomitant treatment of P19 and F9 embryonal carcinoma cells with RARα and RARγ agonists at receptor-selective concentrations favoured differentiation, whereas each agent when used alone failed to promote differentiation. Crosstalk with other signalling events is important, for example, fibroblast growth factor (FGF) for ES cells and the lineage instructive haematopoietic cytokines.

There are additional controls on decision-making for differentiation that include signalling events that are provoked by, for example, FGF for ES cells and the hematopoietic cytokines that instruct cell lineage (Figure 2). The importance of cytokines is emphasised by studies of HSCs derived from ES cells. Human HSCs (CD34^+^) cells were efficiently derived from ES cells by coculture with OP9 bone marrow stromal cells. When isolated cells were cultured on MS-5 stromal cells with the addition of stem cell factor, Flt-3 ligand, interleukin 7 (IL-7), and IL-3 they generated granulocytes, macrophages, B-cell, and natural killer cells (Vodyanik et al., 2005). Similarly, the treatment of human ES cells with a combination of cytokines and bone morphogenic protein-4 promoted the differentiation of hematopoietic progenitors. The cells generated included colony-forming units for granulocytes, macrophages, and erythrocytes together with multipotent colony-forming units (Chadwick et al., 2003).

## RARα and RARγ controls on decision making and cancer

Cancer is a decision-making process whereby cancer stem cells (CSCs) generate the hierarchy of developing cells to sustain a cancer (Dick, 2008). CSCs appear to arise largely from the malignant transformation of a tissue-specific stem cell and are, therefore, immortal [reviewed in Brown, 2022]. Often, the progeny of CSCs undergoes partial differentiation and belongs to a cell lineage; cancers are categorized according to the resemblance of the bulk cells to a cell type. Support to the lineage restriction of the progeny of CSCs has been provided by the findings from transgenic mice whereby restriction of an oncogenic insult to HSCs/haematopoietic progenitors led to restriction of the lineage options of CSCs or introduced a bias (Gonzales-Herrero et al., 2018).

A role for the fusion gene PML-RARα in the pathogenesis of acute promyelocytic leukaemia is well established (de The et al., 1990). For nine acute promyelocytic patients, fusions have been described between RARγ and the genes for PML, NUP98, CPSF6 and NPM1, and these patients failed to respond to ATRA except for the one patient with the PML-RARγ fusion (Conserva et al., 2019). A recent global study identified 34 patients with RARγ rearrangements, the partner genes were diverse, and the rearrangement conferred a poor prognosis (Zhu et al., 2023).

From the importance of RARγ to decision-making by stem cells, we might expect RARγ to be an oncogene. RARγ overexpression has been reported for good proportions of patients with cholangiocarcinoma, clear cell renal cell carcinoma, colorectal cancer, ovarian cancer, and pancreatic ductal adenocarcinoma. For cholangiocarcinoma, overexpression was associated with poor differentiation and metastasis to lymph nodes and contributed to multidrug resistance. Findings suggested that the role of RARγ is mediated *via* activation of the Akt/NFκB and Wnt/B-catenin pathways and upregulation of P glycoprotein (Huang et al., 2013). RARγ and RARβ were upregulated in clear renal cell carcinoma, as seen from a bioinformatics analysis and the use of quantitative PCR (Kudryavtseva et al., 2016). For colorectal cancer, RARγ overexpression was linked to multidrug resistance with knockdown leading to downregulation of multi-drug resistance 1 and suppression of the Wnt/β-catenin pathway (Huang et al., 2017). High expression on ovarian cancer was a predictor of poor overall survival outcomes and has been linked to accelerated disease progression *via* the regulation of cell proliferation (Xiu et al., 2022). Overexpression of RARγ in pancreatic ductal adenocarcinoma tissue and high-grade precancerous lesions was linked to a poor patient prognosis and blocking RARγ signalling supressed the proliferation of cancer cells (Yamakowa et al., 2022). For the above carcinomas, a common denominator was that a high level of expression of RARγ was linked to a poor prognosis. Targeting RARγ to treat disease is a promising prospect because for prostate cancer, agonism of RARγ stimulated the growth of and colony formation by prostate cancer cell line cells and antagonising led to necroptosis of the cell line colony forming CSC-like cells and patients’ cells (Petrie et al., 2022). The pan-RAR antagonist AGN194310 was also effective in ablating the formation of neurosphere-like structures by the CSCs of two paediatric patients’ primitive neuroectodermal tumours and a paediatric patient’s astrocytoma and killed the progeny of CSCs (Brown and Petrie, 2012). The extent to which increased expression of RARγ and imbalance to the levels of expression of RARγ and RARα had led to the development of the above carcinomas by deregulating the behaviour of CSCs is still unclear. Overexpression of RARγ may play a role to maintain CSCs or to restrict these cells to a particular cell lineage. The latter is an intriguing consideration in view of the role of RARγ2 in ES neuronal differentiation and perhaps the need to eliminate other fates (Ying et al., 2003), agonising RARγ interfered with patterning for zebrafish development (Wai et al., 2015), and the need for RARγ for chromatin epigenetic marks (Kashyap et al., 2013). Regarding epigenetic marks, RARγ was identified as the predominant mediator of ATRA-mediated signalling within ES cells for activation of the *Hoxa* and *Hoxb* gene clusters. It was required for broad epigenomic organisation and necessary for the gene-specific removal of the polycomb repressive mark H3K27me3 during ES cell differentiation. *Hox* gene cluster reorganisation was triggered by RARγ located at the *Hoxa1* 3´-RARE and deletion of the RARγ binding site within the *Hoxa1* enhancer attenuated epigenomic activation of *Hoxa* and *Hoxb* gene structures (Kashyap et al., 2011).

## Perspectives and conclusion

Distinct ATRA gradients and boundaries, from cells that synthesize and metabolize, are important to patterning embryogenesis, but there does not appear to be a linear relationship between dose and phenotype (Bernheim and Meilhac, 2020). Gradients and the dose of ATRA that stem cells and their progeny are exposed is relevant to decision-making because sub nM ATRA is sufficient to activate RARγ with RARα activation needing substantially more. Stem cells are equipped to store and degrade ATRA and seem unable to synthesize which may provide protection from ATRA-modulation of the frequency of decision-making for differentiation. RARγ activation is important to the maintenance of HSCs and their stemness and activation, in the absence of active RARα, interfered with decision-making by zebrafish embryonic stem cells for differentiation. The level of ATRA that is used routinely to drive the differentiation of ES cells and other cells, which express both RARγ and RARα, activates both RARα and RARγ and concomitant or temporal activation of these receptors favors differentiation. Stem cell differentiation is a continuous and progressive process which might favour a temporal interaction(s), but whether concomitant or temporal is yet unclear. Expression of RARγ decreases as stem cells differentiate leaving RARα to exert a sole influence on the progression of differentiation.

The roles of RARα and RARγ are modulatory, rather than obligatory, which raises the question what is nature of the events that are being modulated. Signalling *via* growth factors and hematopoietic cytokines provide a further input to ES cell and HSC decision-making for cell differentiation, respectively. That stem cell differentiation is a progressive and gradual process presumably requires learning and memory of the events that are provoked by growth factors/cytokines. There is evidence to support integration of ATRA signalling with how cells learn from the events that are provoked by growth factors because CRABP1 delivers ATRA to the CYP26 family members for degradation and such dampens the sensitivity of ES cells to growth factors to affect their learning and memory (Nagpal and Wei, 2019). An intriguing possibility is whether RARα and/or RARγ are modulatory by virtue of influencing the retention or loss of cell learning and memory for stemness and decision-making for differentiation. The epigenome has been proposed as the judge, jury, and executioner of stem cell fate (Tollervey and Lunyak, 2012). As above, RARγ is needed for ATRA-induced chromatin epigenetic marks [Kashyap et al., 2012], and memory/learning are written within the epigenome by marks.

Presently, we know how RARs work as heterodimers with RXRs, how they bind to response elements, that they repress gene expression in the absence of ligand and drive expression when ligand is bound, and that the RAR subtypes can regulate sub-sets of genes. However, the more complete picture regarding how all of this modulates the specification of cell lineage and/or the switch from stem cell maintenance to the onset of differentiation is still a complex and unresolved puzzle. Considerations include the extent to which ATRA is synthesized endogenously, which though cryptic is at a physiological level, the provision of ATRA by neighbouring cells including from gradients, which is again physiological, whether RARα and/or RARγ are activated, and crosstalk with other cytokine-provoked signalling cascades including phosphorylation of RARs.
